# Integrin targeting of glyphosate and its cell adhesion modulation effects on osteoblastic MC3T3-E1 cells revealed by label-free optical biosensing

**DOI:** 10.1038/s41598-018-36081-0

**Published:** 2018-11-27

**Authors:** Inna Szekacs, Eniko Farkas, Borbala Leticia Gemes, Eszter Takacs, Andras Szekacs, Robert Horvath

**Affiliations:** 1 Nanobiosensorics Momentum Group, Institute of Technical Physics and Materials Science, Centre for Energy Research, Hungarian Academy of Sciences, Konkoly-Thege M. út 29–33, H-1120 Budapest, Hungary; 2 Subdoctoral School of Molecular and Nanotechnologies, Chemical Engineering and Material Science Doctoral School, University of Pannonia, Egyetem u.10, H-8200 Veszprém, Hungary; 3 Agro-Environmental Research Institute, National Agricultural Research and Innovation Centre, Herman Ottó u. 15, H-1022 Budapest, Hungary

## Abstract

This study is a discovery of interesting and far reaching properties of the world leading herbicide active ingredient glyphosate. Here we demonstrate the cell adhesion-modifying characteristics of glyphosate affecting cellular interactions via Arg-Gly-Asp (RGD)-dependent integrins. This conclusion was supported by the observations that a glyphosate surface coating induced integrin-specific cell adhesion, while glyphosate in solution inhibited cell adhesion on an RGD-displaying surface. A sensitive, real-time, label-free, whole cell approach was used to monitor the cell adhesion kinetic processes with excellent data quality. The half maximal inhibitory concentration (IC_50_) for glyphosate was determined to be 0.47 ± 0.07% (20.6 mM) in serum-free conditions. A three-dimensional dissociation constant of 0.352 mM was calculated for the binding between RGD-specific integrins in intact MC3T3-E1 cells and soluble glyphosate by measuring its competition for RGD-motifs binding, while the affinity of those RGD-specific integrins to the RGD-motifs was 5.97 µM. The integrin-targeted affinity of glyphosate was proven using competitive binding assays to recombinant receptor αvβ3. The present study shows not only ligand-binding properties of glyphosate, but also illustrates its remarkable biomimetic power in the case of cell adhesion.

## Introduction

Cell adhesion is the fundamental process in tissue development by which cells form contacts with each other or with their substratum through specialized protein complexes. Although cells express various cellular adhesion molecules (such as cadherins, members of the immunoglobulin superfamily, syndecans, integrins, and selectins), the integrin transmembrane heterodimeric receptors are the most studied family and play an important role in cell–cell and cell–extracellular matrix (ECM) interactions. Divergence of the integrin subunits provides a basis of their versatility in initiating cell adhesion processes^1^. Certain integrins are quite specific in their ligand-binding properties for the common Arg-Gly-Asp (RGD) tripeptide sequence of the ECM proteins. Integrin–ligand interactions activate many critical signal transduction pathways. Therefore, targeting of integrins may interfere with normal cellular functions and play critical roles in modulating cellular processes including proliferation, migration, differentiation and survival^2^.

Toxicants can affect cellular processes through receptors, ion channels, enzymes, binding proteins or cytoskeleton molecules and thus may alter normal functioning of the cell. Different xenobiotics can cause a wide variety of biological effects, acute toxicity, immunological reactions, disturbances in the hormonal homeostasis through non-genotoxic mechanisms^3,4^ or cancer through genotoxicity^5^. Several studies have showed impacts of xenobiotics on cellular signalling, cell plasticity, adhesion and migration^6^, and due to its expanding use as an agricultural and household herbicide, glyphosate (N-(phosphonomethyl)glycine) has come into the focus of toxicity studies. Although glyphosate is an organophosphonate, similarly to organophosphate insecticides, has been shown to undergo enzymatic biodegradation e.g. by microorganisms including *Agrobacteria*^7,8^. Numerous studies have indicated *in vitro* toxicity of glyphosate and its formulated products on various cells, as well as *in vivo* toxic effects on a wide range of organisms from ecotoxicity indicator organisms to man. Recent studies showed cytotoxicity of glyphosate on various cell lines including human fibroblast (GM38) and human fibrosarcoma (HT1080) cells^9^, human epithelial type 2 (HeLa contaminant) cells (Hep-2)^10^, embryonic kidney (HEK293) and human hepatoma (HepG2) cells^11^, human epithelial keratinocyte cells^12^, human choriocarcinoma (JEG3) cells^11,13^, NE-4C: murine stem cell-like neuroectodermal cells^14^, human chorioplacental (JAr) cells^15^, human hematopoietic Raji cells (Epstein-Barr virus transformed human lymphocytes)^16^, and murine osteoblastic cell line (MC3T3-E1)^17^.

Exposure of rat hippocampal pyramidal cells to glyphosate at 2–6 mg/ml caused neuronal abnormalities^18^, and glyphosate absorption across Caco-2 epithelial cell tissues indicated neurotoxicity-related saturable glyphosate uptake through epithelial transporter enzyme activity in an ATP- and Na^+^-independent manner, not competed by specific amino acids or transporter inhibitors^19^. At concentrations of 0.09–1.7 mg/ml it caused DNA damage in leucocytes such as human peripheral blood mononuclear cells, and trigger DNA methylation in human cells^20^. It also showed inhibition of aromatases, key enzymes in steroid hormone biosynthesis^21^, and its teratogenic effects on vertebrates were linked to the retinoic acid signaling pathway^22,23^. Moreover, glyphosate-based herbicides exerted even stronger toxicity e.g., Roundup Transorb caused thyroid hormone homeostasis imbalance in male rats^24^.

Currently, cytotoxicity studies are based mainly on conventional end-point methods with long preparation and incubation procedures, many of them are using labels and easily confined by high cost and low-throughput. Development of biosensor techniques and their application work out in different areas, including cytotoxicity studies, is becoming of growing significance. Especially, whole cell-based sensors become extremely important due to their possibility to measure comprehensive and functional effects of different xenobiotics. Biosensors, as rapid, sensitive, and low-cost screening techniques, are applicable in clinical diagnosis and in monitoring of environmental pollutants as well. In the past years, the evanescent filed-based surface sensitive resonant waveguide grating (RWG) biosensor Epic BenchTop (BT) has been proven as a useful method for real-time, high-throughput, and label-free detection of cell adhesion, spreading and signalling events based on measuring of dynamic mass redistribution within a 150 nm range on the sensor surface^25–28^.

Recently, we suggested an approach for the feasibility of using the RWG technology for the analysis of integrin–ligand interactions by measuring the kinetics of cell adhesion^29^. The proposed fast and non-invasive screening tool uses intact cells, is applicable for label-free screening of potential pharmaceutical compounds, and it can also be useful in studying the effects of xenobiotics on cell adhesion processes. In this approach, a change in the resonant wavelength of the guided light occurs when cells adhere and spread on the sensor surface until an adhered cell monolayer is formed. A signal is detected as a shift in the resonant wavelength (Δ*λ*), the magnitude of which is proportional to the area of the sensor covered by spread cells and the local refractive index increment inside the evanescent field^29–31^. Cell adhesion molecules and complexes are located in the evanescent field ~150 nm above the sensor surface, thus contribute to the local refractive index shift. Therefore, non-adhering cells, which are located in the bulk assay medium outside of the evanescent field, are not detected by the biosensor and are excluded from the measurement. In our previous study^17^ on adhered MC3T3-E1 cells we found that the shape of glyphosate-treated cells became elongated and glyphosate caused cell detachment by a yet undeciphered mechanism. A similar effect was reported by Elie-Caille and co-workers on a HaCaT keratinocyte cell line^32,33^. On the basis of our previous results we assume that the mechanism of these induced changes in cell morphology are likely to progress through interaction with cell integrin receptors. In the present study, we examine the impact of glyphosate on cell adhesion processes by using the Epic BT technique and assess differences in its affinity to RGD-specific integrins in intact MC3T3-E1 cells compared to those of RGD-motifs.

## Results and Discussion

Toxic effects of environmental chemicals can lead to passive (necrosis) or active (apoptosis) cell death, and the integrated cellular events of which, induced by given xenobiotics, are readily detected with biosensor techniques. In our previous study^17^ addition of glyphosate at concentrations of 0.4% and above caused complete detachment of entire MC3T3-E1 cell layers from the sensor surface, while lower concentrations resulted in modified cell morphology of adhered cells on the surface. These attachment-modifying characteristics indicate influences on adhesion processes possibly though affecting cellular interactions with cell-adhesion proteins. However, glyphosate has not been reported previously to act as a ligand for integrins. To confirm that glyphosate behaves as a cell integrin ligand, we performed cell adhesion assays on RGD-displaying polymer (well-known to promote cell adhesion) and glyphosate surfaces using the Epic BT technique.

### Adhesion on RGD density tuned surfaces and integrin–RGD dissociation constant in living MC3T3-E1 cells

To characterize the adhesion of MC3T3-E1 cells on RGD-displaying biomimetic surfaces, the dependence of the kinetics of the adhesion process on the surface density of RGD was determined using RGD surface density tuned sensor surfaces. Table 1 summarizes the calculated data for the copolymer mixtures used for RGD-tuning, regarding the estimated molar surface density of the RGD ligands, ligand-to-ligand distances, and numbers of ligands per unit area in each composition applied. The ratio of the RGD moieties on the surface was adjusted using a mixture of poly(L-lysine)-*graft*-poly(ethylene glycol) (PLL-*g*-PEG) and PLL-*g*-PEG-RGD. The composition of the PLL-*g*-PEG component was PLL(20)-*g*(3.5)-PEG(2) (hereafter PP), while that in the RGD-containing component was PLL-*g*-PEG/PEGGGGGYGRGDSP (PLL-*g*-PEG-RGD-12% [PLL(20)-*g*(3.5)-PEG(2.3)/PEG(3.4)-RGD]) (hereafter PPR). Thus, the copolymer PP:PPR was composed of several molecular moieties of different molecular weights (MWs): PLL (MW: 20 kDa) grafted with short non-functionalized PEG (MW: 2 kDa) and long PEG (MW: 3.4 kDa) terminated with RGD-motif ^34^. The molecular weight of the copolymer (MW_PP:PPR_) was calculated using following equation^35^:

1 $$M{W}_{PP:PPR}=M{W}_{PLL}+\frac{M{W}_{PLL}}{M{W}_{Lys}}\frac{1}{g}[(1-P)M{W}_{PEG}+P\cdot M{W}_{PPR}],$$

where P = 12% is the fraction of functionalized PEG per copolymer. Thus MW_PP:PPR_ was determined to be 88.4 kDa. The surface density of the RGD-motif (ρ_RGD_) was calculated from copolymer composition^26,36^:

2 $${\rho }_{RGD}=\frac{\Gamma }{M{W}_{PP:PPR}}\frac{Q}{100}\frac{{N}_{Lys}}{g}P,$$

where *Γ* is the mass of the copolymer adsorbed to the Nb_2_O_5_ sensor surface, reported to be 184 ng/cm^2^^34^, Q is the volume percent of the PPR moieties in the mixture with PP, N_Lys_ = 96 is the average number of Lys units per PLL backbone, and g = 3.5 is the number of Lys units per PEG chain (grafting ratio). The estimated ligand-to-ligand distance (d_RGD–RGD_), assuming hexagonal distribution of the ligands on smooth surface^36^, is obtained from:

3 $${d}_{RGD-RGD}=\sqrt{\frac{2}{\sqrt{3}}\frac{1}{{\rho }_{RGD}\cdot {N}_{A}}},$$

where N_A_ = 6.022 × 10^23^ mol^−1^ is Avogadro’s number. The numbers of ligands per unit area (ν_RGD_) were calculated from ligand surface density (ρ_RGD_) by multiplying it with Avogadro’s number.

**Table 1:** Volume percent (Q) of 1 mg/ml PPR in the mixed solutions of copolymers and calculated subsequent molar surface density of RGD-motifs (ρ_RGD_), ligand-to-ligand distance (d_RGD–RGD_), and numbers of ligands per unit area (ν_RGD_).

Q (%)	ρ_RGD_ (pmol cm^−2^)	d_RGD–RGD_ (nm)	ν_RGD_ (μm^−2^)
0	0	∞	0
0.125	0.0086	150	52
0.25	0.0171	106	103
0.5	0.0343	75	206
1	0.0685	53	413
2	0.1371	37	825
4	0.2742	26	1651
10	0.6854	17	4127
25	1.7135	11	10319
50	3.4270	8	20637
100	6.8539	5	41274

MC3T3-E1 adhesion appeared to proportionally increase with the RGD-motif surface density, ρ_RGD_. This process occurred to be slightly affected by preosteoblast cells showing a limited adherence capability to the non-adhesive PP surface. Such non-specific adhesion to the assumedly protein-repellent PP surface has been reported for several cell types, including osteoblast cells^36^, and has been attributed to incomplete surface coverage by PP due to small defects in the PEG-brushes. To assure that adhesion assessments reflect only integrin-specific binding to RGD-motifs in the calculations, the background due to non-specific binding to PP was subtracted from the biosensor responses.

**Figure 1:**
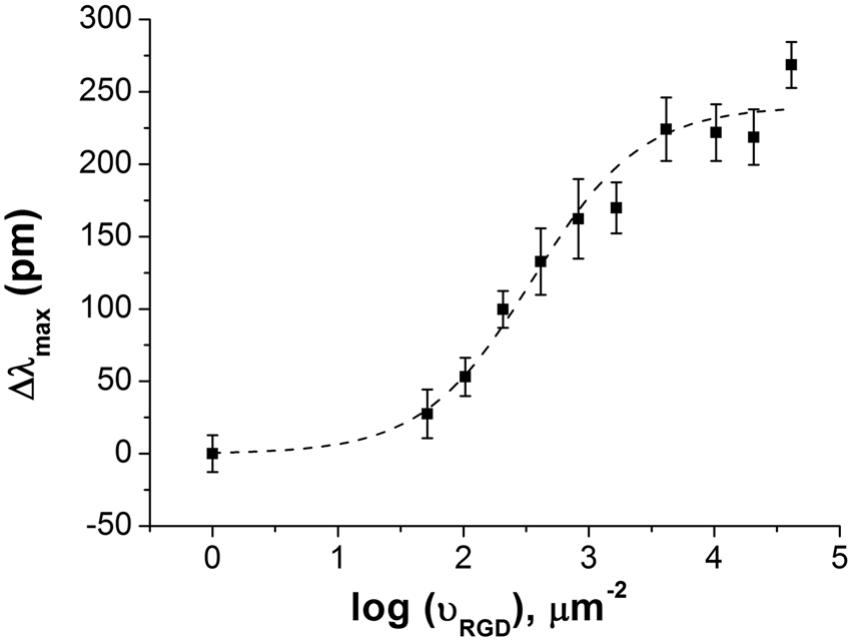
Ligand concentration-dependence of the biosensor response. The curve indicates a two-dimensional dissociation constant (^2D^K_d (PPR)_) for the binding between PPR and RGD-specific integrins embedded in the cell membrane to be 359.6 ± 62.1 µm^−2^. Each data point represents the mean ± standard deviation (SD) of the average value of data obtained in single experiment, in triplicates (n = 3).

The two-dimensional dissociation constant for integrin-RGD binding (^2D^K_d_) was obtained from the fitting of the biosensor signal at saturation level (Δλ_max_) as a function of the average number of ligands per unit contact area (ν_RGD_). Thus, in logistic non-linear fitting, supposing the binding of a given integrin ligand proceeds on a single integrin binding site as an equimolar process and there is no cooperativity for the binding of ligand^26,37^, ^2D^K_d (PPR)_ was found to be 359.6 ± 62.1 μm^−2^ with an adjusted R^2^ value of 0.972 (Fig. 1).

The ^2D^K_d_ constant provides information about the equilibrium of the surface-bound ligand and intact cell integrin as a planar adhesion process. It can be easily converted to a three-dimensional dissociation constant (^3D^K_d_) in solution^38^:

4 $${}^{3D}{K}_{d}=\frac{{}^{2D}{K}_{d}}{h},$$

where *h* is the confinement length^39^, representing the cell–substrate separation distance of 100 nm^26^. The estimated value of ^3D^K_d (PPR)_ for the affinity of the RGD-specific integrins in the intact MC3T3-E1 cells and RGD-motifs was found to be 5.97 µM. This value shows nearly 5-fold higher affinity for RGD-motifs to RGD-specific integrins of embedded in the membrane of MC3T3-E1 cells, than that previously reported for HeLa cells^26^. We believe, this is in connection with the type of integrins expressed by the two cell types, being a more diverse population in case of the preosteoblast cells^40–42^.

### Cell adhesion assay on glyphosate-containing surfaces – comparison to the adhesion on RGD-containing surface

**Figure 2:**
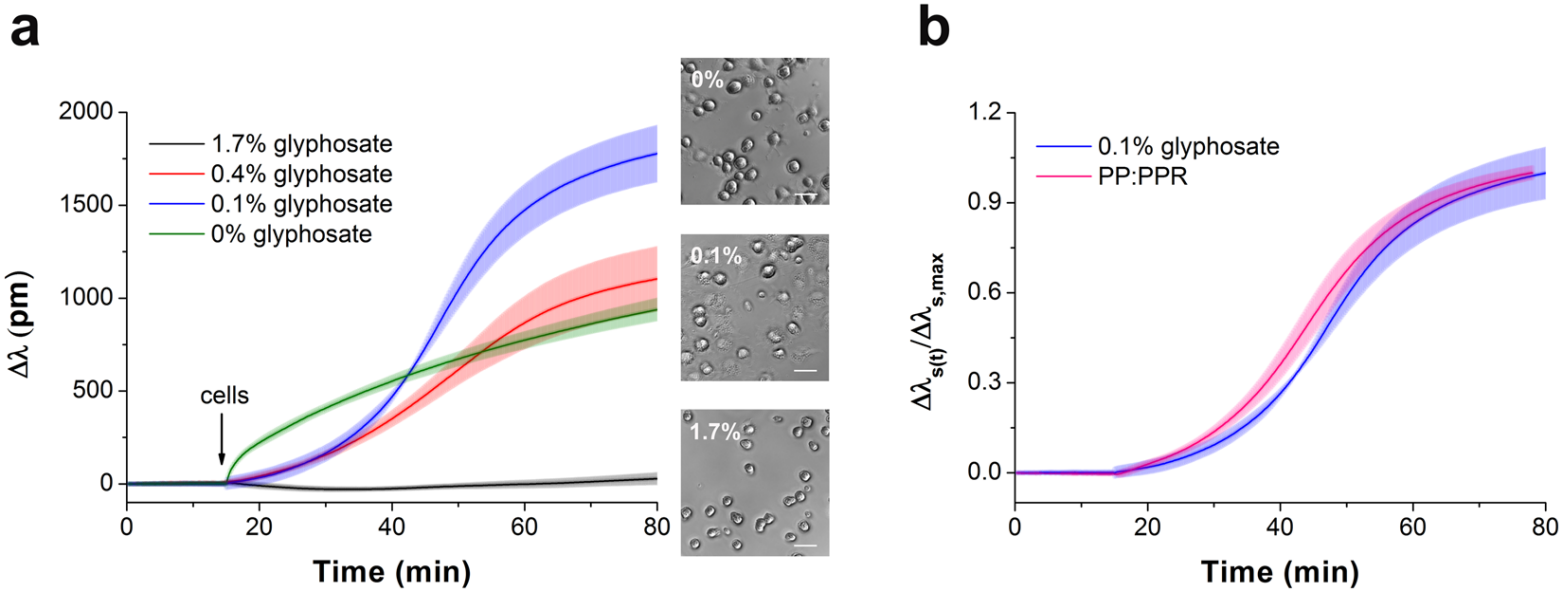
Glyphosate-provoked adhesion of MC3T3-E1 cells in 20 mM HEPES HBSS, pH 7.4. (**a**) Biosensor signals recorded during MC3T3-E1 cell addition onto the surface containing different concentrations of glyphosate. Data represent the mean ± SD (clouds around the curves) of the data obtained in triplicates (n = 3). The phase contrast images (scale bar: 50 μm) show cells on the biosensor surfaces right after the Epic BT measurements. (**b**) Kinetics of MC3T3-E1 cell adhesion to 0.1% glyphosate (*blue*) and PP:PPR (*pink*).

The kinetics of MC3T3-E1 cells adhesion in the wells of the Epic cell assay microplate (Fig. 2) indicate a considerable effect of glyphosate on the cell adhesion process. It is known, that cell adhesion to an uncoated sensor surface under serum-free conditions is a non-specific process showing adsorption-like kinetics (Fig. 2a, green curve). Surprisingly, at a concentration of 0.1% glyphosate enhanced cell adhesion compared to the control cells, and in the case of its low concentrations (0.1–0.4%) a sigmoid-like response was obtained (Fig. 2a). Note, the sigmoidal shaped kinetics is a typical characteristics of receptor mediated cell adhesion events^25,26^. Microscopy images also confirm that cells adhered to the surface treated with glyphosate had more spread shape than the control cells on uncoated surface (Fig. 2a). Normal cell structures were detected in control cells by phase contrast microscopy, while notable vacuolization was found in the cells on glyphosate-containing surface.

As evidenced by the experiments, cell adhesion is gradually suppressed at higher glyphosate concentrations (0.4–1.7%) (Fig. 2a, red and black curves). This phenomenon, manifested in the damping of the sigmoid curve, is likely to be caused by sensor surface saturation with glyphosate and subsequent increasing occurrence of the compound in the solution. This effect leads to a blockage of cell adhesion probably due to non-adsorbed glyphosate. At a concentration of 1.7% no cell adhesion signal was detected (Fig. 2a, black curve).

Based on this data, we suppose that adsorbed glyphosate reproduces a biomimetic surface and enhances cell adhesion. Nevertheless, glyphosate molecules in solution effectively block the integrin receptors of the cells and decrease cell adhesion ability. To verify this hypothesis, (*i*) the effects of surface-bound glyphosate on cell adhesion were compared to that of the known adhesion motif RGD; (*ii*) the strength of binding of glyphosate to the sensor surface was tested; and (*iii*) the effect of soluble glyphosate at increasing concentrations on cell adhesion were assessed (see below) on RGD displaying polymer surfaces.

Comparing the biosensor signal obtained upon cell adhesion to 0.1% glyphosate coating with that to an RGD-motif displaying surface (knowing to promote cell adhesion) indicates that the two processes show similar, almost identical kinetics (see Fig. 2b). Biosensor responses were normalized to the maximal signal obtained on the biosensor surface covered with the given substance: Δλ_s_(*t*)/Δλ_s,max_, where Δ*λ*_s_(*t*) indicates the actual change in the resonant wavelength of the guided light belonging to cell adhesion to the given substance on the surface (*s*) and time point (*t*), and Δλ_s,max_ indicates the maximal wavelength shift recorded for cell adhesion with the given substance on the biosensor surface. The results support the new concept that glyphosate may act, by a yet unidentified mechanism, as an integrin ligand that promotes surface adhesion of cells.

To compare binding affinity to integrin, molecular amounts of glyphosate and RGD-motifs in PP:PPR adsorbed onto Nb_2_O_5_ biosensor surface were liken. In the optimized surface modification protocol 500 μg/ml PP:PPR was applied, corresponding to the RGD molar surface density of 3.4270 pmol/cm^2^ and 20637 ligands/μm^2^ (see Table 1). The quantity of surface adsorbed glyphosate in case of 0.1% (that results in a sigmoid curve, technically identical to that obtained with 500 μg/ml of PP:PPR) without washing was measured to be 4 ng/cm^2^, corresponding to a molar surface density 17.53 pmol/cm^2^ and 105180 ligands/μm^2^. Based on these results an average intermolecular distance between glyphosate molecules was calculated to be 3.3 nm by using Eq. 3. This surface density is completely in line with the above hypothesis, an averaged RGD–RGD distance below 10 nm leads to strong sigmoidal shaped cell adhesion^26,36^, and maximum spacing between the ligands to induce cell adhesion was reported to be about 70 nm for MC3T3 cells^43^. Moreover, the biosensor surface may facilitate functional orientation of glyphosate molecules involving cation binding thus, stabilizing glyphosate in more efficient position for cell adhesion.

**Figure 3:**
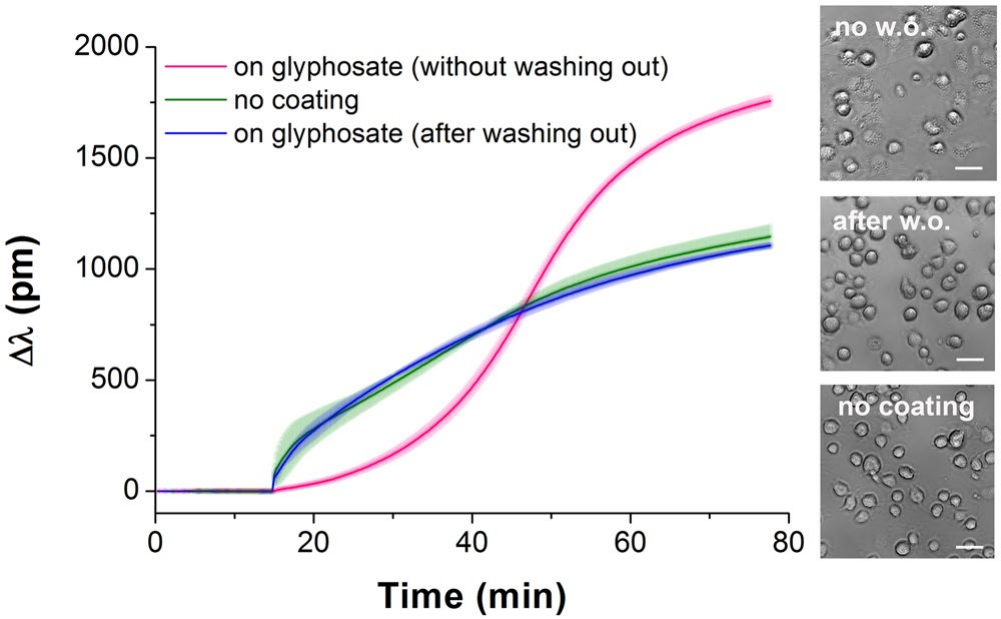
Kinetics of MC3T3-E1 cell adhesion on sensor surfaces with various coating conditions. No surface coating (*green*); wells incubated with 0.2% glyphosate for 30 min and then rinsed three times with buffer before adding the cells (*blue*); cell adhesion to 0.1% glyphosate coating (*pink*). Data represent the mean ± SD (clouds around the curves) of the data obtained in triplicates (n = 3). The phase contrast images (scale bar: 50 μm) show cells on the biosensor surfaces right after the Epic BT measurements.

To check if glyphosate could be irreversibly adsorbed on the surface, it was incubated on the sensor surface for 30 min at room temperature, and then the surface was rinsed three times with 20 mM HEPES HBSS, pH 7.4. In this case the sensor response to cell adhesion showed adsorption-like kinetics (almost identical to the signal on the uncoated surface) indicating that glyphosate has predominantly been washed off from the sensor surface with intense rinsing (Fig. 3).

### Cell adhesion inhibition assay and integrin–glyphosate dissociation constant in living MC3T3-E1 cells

**Figure 4:**
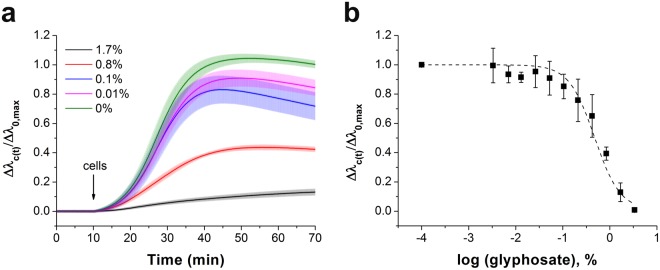
The effect of glyphosate on MC3T3-E1 cell adhesion onto PP:PPR surface in the Epic BT assay. (**a**) Typical spreading curves obtained after MC3T3-E1 cells were exposed by preincubation to glyphosate at different concentrations. (**b**) Concentration-dependence of the biosensor response indicating a half maximal inhibitory concentration (IC_50_) of 0.47 ± 0.07% (20.6 mM). Each data point represents the mean ± SD of the average value of data obtained in four experiments, in triplicates in each experiment (n = 12), for each concentration.

The whole cell Epic BT biosensor approach to determine the affinity of ligands to cell adhesion receptors^26^, developed and applied previously to identify the half maximal inhibitory concentration (IC_50_) for echistatin^29^, was employed here to detect the cell adhesion process under the impact of glyphosate. First, the sensor surfaces were coated with an RGD-motif-displaying PP:PPR film. Then, MC3T3-E1 cells were preincubated with glyphosate at different concentrations, these suspensions were pipetted onto the sensor surfaces, and inhibition of the cell adhesion process was determined using the Epic BT instrument. All biosensor responses were normalized to the maximal signal of the negative control (Δ*λ*_c_(*t*)/Δ*λ*_0,max_, where Δ*λ*_c_(*t*) indicates the actual change in the resonant wavelength of the guided light belonging to the given glyphosate concentration (*c*) and time point (*t*), and Δ*λ*_0,max_ indicates the actual change in the resonant wavelength of the guided light belonging to 0 glyphosate concentration (0) at t > 60 min of cell adhesion). The results illustrate a dose-dependent decrease in cell adhesion in response to preincubation of the cells with glyphosate (Fig. 4). In the presence of an inhibitor, cell adhesion is decreasing due to integrin blocking^29^, resulting in reduction in the maximal biosensor signal (Fig. 4a). The flat kinetic curve indicates that the spreading of MC3T3-E1 cells was completely blocked by preincubation with 1.7% glyphosate. The Epic BT signals in the different measurements were analysed and compared by normalizing data of each single well to the appropriate negative control well (Fig. 4b). The half maximal inhibitory concentration (IC_50_) value was calculated as a combined value from four separate experiments.

The binding between RGD-specific integrins in the intact MC3T3-E1 cells and soluble glyphosate is a competition at a single site for RGD-motifs binding. The estimated value of the ^3D^K_d (glyphosate)_ for the binding of cell integrins and glyphosate was calculated to be 0.352 mM by using equation from Cheng and Prusoff^44^:

5 $${}^{3D}{K}_{d(glyphosate)}=\frac{I{C}_{50}}{1+\frac{[RGD]}{{}^{3D}{K}_{d(PPR)}}},$$

where IC_50 (glyphosate)_ is the half maximal inhibitory concentration for glyphosate (20.6 mM), [RGD] is the concentration of RGD on the sensor surface (assuming a confinement length of 100 nm for the RGD moieties yields [RGD] = 0.343 mM), and ^3D^K_d (PPR)_ is the dissociation constant in solution for the binding between PPR and RGD-specific integrins embedded in the cell membrane, its value obtained for MC3T3-E1 cells is 5.97 × 10^–3^ mM (see Eq. 4).

Moreover, the above determined dissociation constants – 352 μM – can be compared to related literature values of dissociation constants of various integrin ligands. The work of Kapp *et al*.^45^ summarizes the most typical integrin ligands and their binding strengths to various integrin types, and discusses the advantages and disadvantages of measurement on isolated integrins. The low affinity binding – K_d_ values larger than 10 μM – is typical for integrin ligands and there are several examples in Table 1 of Kapp *et al*.^45^. Therefore, the obtained value suggests the possibility of biological significance. Considering the kinetic parameters of the binding, such a value would result in a k_off_ range of 10^3^–10^5^ s^−1^ with typical k_on_ values of 10^6^–10^8^ M^−1^s^−1^ according to the calculations of Sanders^37^, considering a typical sized ligand.

### The explanation of the observed effects of glyphosate on cell adhesion

**Figure 5:**
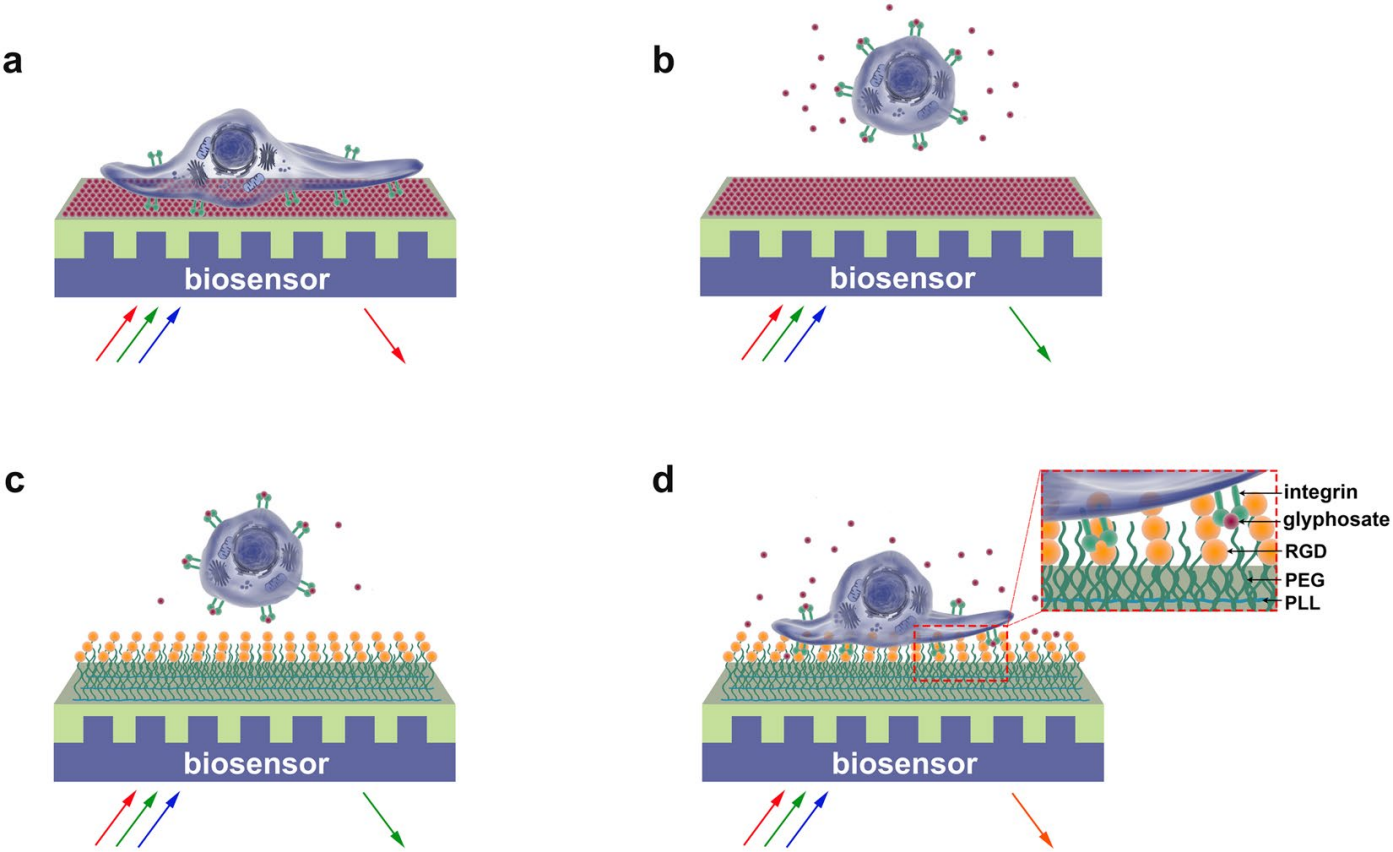
Schematic illustration of the working principle of the cell-based biosensor and the concentration-dependent effects of glyphosate on cell adhesion. (**a**) MC3T3-E1 cells spread on a sensor surface treated with 0.1% glyphosate solution. (**b**) Inhibition of cell adhesion by glyphosate at 0.2–1.7% concentration in the solution (with complete blockage achieved at 1.7%). (**c**) Prevention of cell adhesion onto PP:PPR surface by preincubation of the cells with glyphosate at 0.47% concentration in the solution. (**d**) Detaching MC3T3-E1 cells adhered to a surface modified with PP:PPR by glyphosate at 0.9% concentration in solution.

The results obtained on the inhibition of cell adhesion by glyphosate explain its concentration-dependent effect observed (see above). At a concentration of 0.1% (4.38 mM) of glyphosate on the bare sensor surface, a sigmoidal shape spreading curve of MC3T3-E1 cells is obtained (see Fig. 2). At this concentration glyphosate molecules saturate the surface and promote cell attachment, and glyphosate molecules still remaining in solution cannot block effectively the adhesion of the cells (see Fig. 5a for schematic explanation). At higher concentrations (0.2–1.7%), however, glyphosate in the solution can suppress cell adhesion, by saturating cell surface integrins (Fig. 5b), even if glyphosate molecules adsorbed to the surface are imitating an RGD pattern (see Fig. 2). At even higher concentrations (IC_50_ = 9.77 mM), glyphosate can drive adhered MC3T3-E1 cells off from the bare sensor surface^17^. This effect is even higher if the MC3T3-E1 cells are preincubated with glyphosate prior to being allowed to come in contact with the surface they could adhere to. Such preincubation with glyphosate at concentrations nearly 5-fold higher (IC_50_ = 20.6 mM (0.47%)) than those promoting cell adhesion can prevent MC3T3-E1 cell attachment even to surfaces activated with PP:PPR containing adhesion promoting RGD moieties (Fig. 5c), as the corresponding IC_50_ value (see Fig. 4) is reached. Moreover, glyphosate at 10-fold higher concentrations (about 40 mM, data not shown) is capable to compete even adhered cells off from a surface modified with PP:PPR (Fig. 5d). Such concentration-dependent dual effects on cell binding processes are seen not only with glyphosate: cell adhesion proteins are also known to be capable to become specific inhibitors of their own function if they were bound in excess to a cellular receptor^46^, and similar concentration-dependent activation and inhibition of cell adhesion has been seen with glycoproteins capable to bind to integrins (fibronectin, vitronectin). Surface coating with fibronectin at 3 μg/ml (7 nM) was effective in attaching cells, while at 3300-fold higher, 10 mg/ml (23 µM) concentrations the same soluble ligands inhibited cell adhesion^46^.

### The role of inhibition of integrins in cell adhesion and the model explaining the observed effects

Integrins are a family of adhesion receptors that interact with a variety of extracellular ligands, typically cell-adhesive proteins in ECM. MC3T3-E1 cells express several integrins, including α2β1, α3β1, α4β1, α5β1, α6β1, αvβ1, and αvβ3^42^. Some integrins are quite specific in their ligand-binding properties for the RGD tripeptide sequence of the ECM. The RGD sequence is the active unit of a large number of adhesive extracellular matrix proteins. It is known that RGD when coated onto a surface promotes cell adhesion, whereas in solution has a blocking effect on adhesion^47,48^. However, the affinity of RGD peptides could be different due to the conformation and nature of the surrounding amino acids, and is significantly (>1000-fold) lower than that of the proteins from which it is derived^49^.

Integrins may play important roles as regulators of apoptosis^50^. Among this inhibition of integrin–ligand interactions, the soluble RGD-based peptides getting to the cell directly may induce apoptosis by direct caspase-3 activation as well^51^. However, there are other types of programmed cell death mechanisms, and cells could undergo anoikis, apoptosis induced by the prevention of cell adhesion^52–54^.

**Figure 6:**
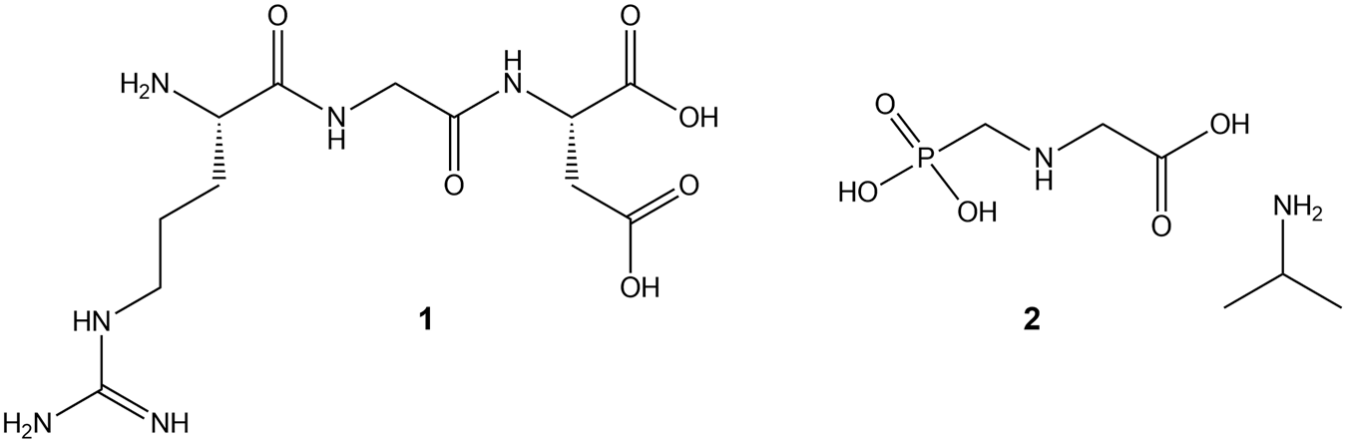
Chemical structures of RGD (**1**) and glyphosate IPA salt (**2**).

Cell adhesion to ECM requires divalent cations in the medium and each integrin heterodimer contains several divalent cation binding sites. The bound divalent cations can moderate the integrin function^55^. The ligand-binding site in the integrins is at or near a binding site for divalent cations^49^. The emulated crystal structures of RGD-liganded αvβ3 show that divalent cations act as a bridge between ligands and the heterodimeric integrin, and the glycine residue makes hydrophobic contacts with αv^56^. The aspartic acid of RGD contributes to divalent cation binding providing one of the coordination sites^57^. Although glyphosate bears no obvious structural similarity to RGD (see Fig. 6), it is a derivative of glycine and is also a strong chelator of Ca^2+^, Mg^2+^, and of many other divalent and trivalent metallic cations^58–60^.

We hypothesize that the carboxy terminus of glyphosate mimics an aspartic acid in RGD-motif and thus the integrin and glyphosate can share the cation. In turn, glycine in glyphosate could make a hydrophobic contact with integrin. Nonetheless, we found that glycine alone could not actually block the integrin receptors or induce cell adhesion at concentrations of 12.5, 25 and 50 mM (data not shown). Loomis *et al*. have observed that glycine at concentrations as high as 50 mM can block *Dictyostelium* cell adhesion through a non-integrin specific mechanism^61^. It should be noted that *Dictyostelium* cells cannot form integrin mediated focal adhesions, unlike mammalian cells, where such interactions generate much stronger adhesion to ECM.

**Figure 7:**
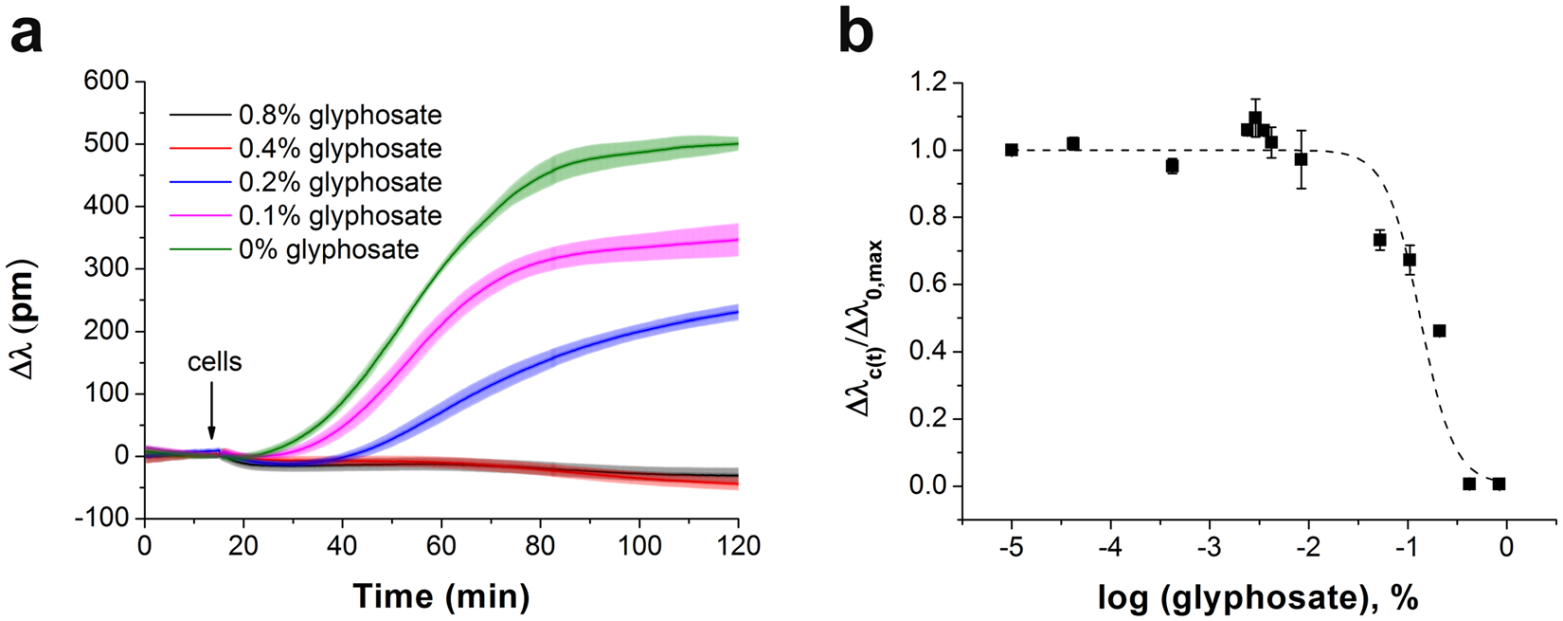
The effect of glyphosate on MC3T3-E1 cell adhesion in serum-containing medium. (**a**) Typical spreading curves obtained after MC3T3-E1 cells were exposed to glyphosate at different concentrations. (**b**) Concentration-dependence of the biosensor response indicating a half maximal inhibitory concentration (IC_50_) of 0.13 ± 0.04% (5.7 mM). Each data point represents the mean ± SD of the average value of data obtained in two experiments, in triplicates in each experiment (n = 6), for each concentration.

These data evidence that glyphosate, under certain conditions, results in integrin blocking, moreover, its differential activity in serum and under serum-free conditions is explained. In our recent publication based on cell detachment measurements, the IC_50_ value for glyphosate obtained in serum-containing medium was reported to be 2.98 mM instead of 9.77 mM under serum-free conditions (in buffer)^17^. From the present methodology, based on measuring the kinetics of cell adhesion, the IC_50_ value for glyphosate in serum-containing medium is 5.7 mM (Fig. 7) in comparison to 20.6 mM of inhibition effect under serum-free conditions. This appeared to be paradoxical, as any toxicant is expected to be less effective in serum-containing medium, as the protein content in serum can attenuate their inhibitory effect. In light of the current results, however, the increased inhibitory potential of glyphosate in serum, compared to serum-free conditions, is explained: serum contains numerous other proteins capable to interact with cellular integrins (e.g. about 220 μg/ml of fibronectin^62^ and 200–300 μg/ml of vitronectin^63^). Their miniscule effects (1/10, 10% FBS is applied for serum-containing medium), however, add up to the inhibitory effect of glyphosate, thus, it can exert effective inhibition at lower concentrations than under serum-free conditions. A vast amount of data in the scientific literature has been published on the differing effects of various synthetic and natural peptides on cell adhesion in serum-containing and serum-free conditions^29,46,47,54,64–72^. A wide variety of the applied concentrations of inhibitors – from nano- to millimolar range – can be explained by not only cell type (and subsequently expressed integrins), surface coating, incubation time, differences in the affinity of inhibitors, but also by presence or absence of serum during experiments.

### Validation of the competitive binding of glyphosate to integrin αvβ3

The integrin-targeting properties of glyphosate was tested in enzyme-linked immunosorbent assay (ELISA) and Epic assay cell-free formats by measuring the competitive inhibition of the binding of a recombinant αvβ3 integrin to the RGD motifs. Integrin αvβ3 is a receptor for vitronectin, nevertheless recognizes a broad range of RGD-containing ECM proteins with different rates of specificity. Snake venom disintegrin echistatin, known to bind to αvβ3 integrin with high affinity, was chosen as a positive control, while tirofiban, with no blocking effect on αvβ3 integrin, was applied as a negative control.

**Figure 8:**
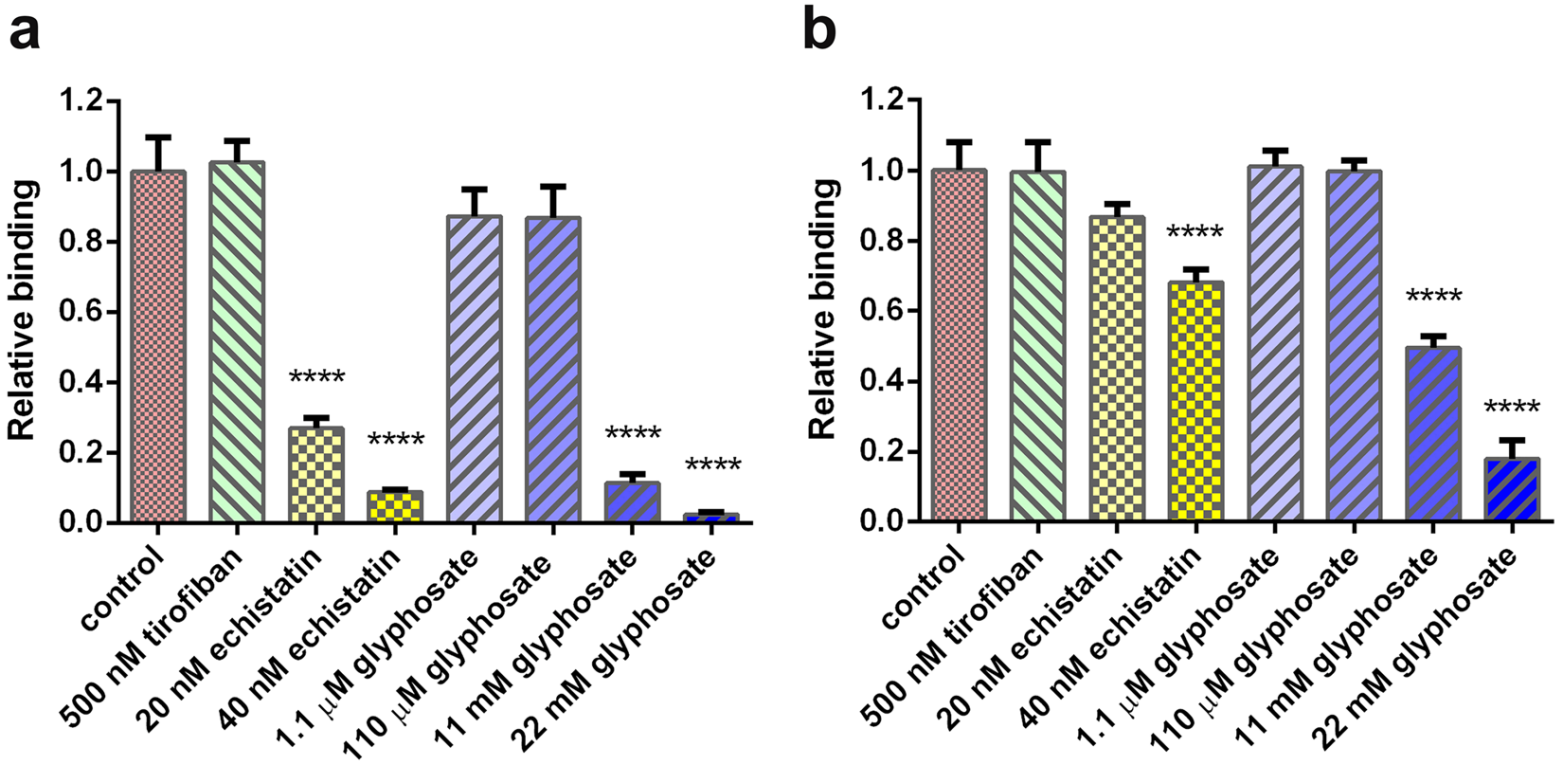
Competitive αvβ3 integrin binding assay with (**a**) ELISA (**b**) Epic BT: Solutions of integrin were co-incubated with the corresponding compounds (glyphosate, echistatin, tirofiban) in the RGD-coated wells of the ELISA or biosensor microplates, and integrin binding to the RGD motifs on the surface was detected. Signals obtained were corrected for the non-specific background (assay signal without integrin or compound addition) and were normalised for assay signals obtained with uninhibited integrin (control). Data are shown as a mean ± SD of at least triplicates (n = 3–6). Statistical analysis was performed with the Graph Pad Prism program. Asterisks indicate significant differences from the control (p < 0.0001).

Data obtained by ELISA and Epic measurements (Fig. 8) clearly indicate concentration-dependent competitive inhibition of the binding of αvβ3 integrin to the immobilised RGD motifs by glyphosate and echistatin, while no significant effects caused by tirofiban. Both techniques revealed the integrin-targeting properties of glyphosate at concentrations at 11 mM and above. The ELISA format appeared to be more sensitive as almost full inhibition was seeen by glyphosate at 11 mM, and substantial inhibition by echistatin at 20 nM. This may be due to the fact that all unreacted components are washed out in the ELISA format, while remain in solution in the sensitive volume and contribute to the assay signal in the Epic assay. In contrast, this slight disadvantage is far compensated in the Epic technique by providing results of the same trends, by not requiring immunoreagents for detection and by its simplicity in the assay protocol.

## Conclusions

From the present study we can conclude several ascertainments. First, MC3T3-E1 cells can adhere to the glyphosate adsorbed on the sensor surface, showing ligand-specific kinetics. Second, soluble glyphosate significantly reduces the MC3T3-E1 cells adhesion via blocking RGD-specific integrins in a concentration-dependent manner, as also validated by cell-free integrin binding assays in both ELISA and Epic formats. Third, it is unknown whether and how glyphosate may interfere with the cellular behaviours provoked by the integrin–ECM interaction in MC3T3-E1 cells.

Based on the results obtained we suggest that glyphosate could interact as a ligand with RGD-specific cell integrins. Glyphosate adsorbed to the surface induced cell adhesion with kinetics similar to adhesion to PP:PPR-modified surface, and a comparison of the ^3D^K_d_ dissociation constants of glyphosate and RGD indicated that approximately 58-fold times more glyphosate molecules than RGD-motifs are required per sensor surface unit to obtain the same cell adhesion kinetics. It means, that glyphosate in a molar surface density of 17.53 pmol/cm^2^ could trigger same cell adhesion kinetic like RGD-motif at 0.3 pmol/cm^2^ (in equivalent to approximately 26 nm RDG–RGD distance). Preincubation of the cells in the presence of increasing concentration of glyphosate in solution resulted in a dose-dependent inhibition of cell adhesion to PP:PPR-modified surface. Indeed, biosensor measurements revealed that glyphosate forms an instable film, it can be completely removed by intense washing with glyphosate free solution, and cell adhesion identical to the adhesion obtained on the bare surface is seen.

Finally, possible physiological consequences of glyphosate being capable to block cell adhesion by mimicking integrin ligands should also be considered. The cytotoxic effects of glyphosate on apoptosis via oxidative stress by forming reactive oxygen species have been widely discussed in the scientific literature. Our results on its interferences with cellular adhesion processes suggest that it may trigger anoikis, as a special form of apoptosis. Such interactions can lead to physiological alternations e.g., bone resorption, abnormal tissue or organ development, decayed immunofunctions or anomalies in processes where cell adhesion plays an essential role.

## Methods

### Chemicals

All chemicals and reagents were obtained from Sigma-Aldrich Chemie GmbH (Schelldorf, Germany), unless stated otherwise. Glyphosate (in form of its isopropylammonium salt) analytical standard used was Pestanal grade, from Riedel-de Haën (Seelze, Germany). A stock solution glyphosate was prepared freshly in 20 mM 4-(2-hydroxyethyl)-1-piperazine ethanesulfonic acid (HEPES) Hank’s balanced salt solution (HBSS) buffer or in serum-containing cell culture medium, the pH was adjusted to pH 7.4, and solution was filtered through a filter (0.22 μm).

### Epic BT biosensor technique

The biosensor measurements were performed using 96- or 384-well Epic cell assay microplates (#5080, #5040, Corning Incorporated, Corning, NY, USA) interrogated by a table top Epic BT instrument (Corning Incorporated, Corning, NY, USA). The bottom of each well of the microplate contains an RWG-based optical biosensor, which consists of a waveguiding layer of Nb_2_O_5_ supported by a corrugated glass substrate. The corrugation acts as an optical grating and incouples the incident light into the waveguide layer, exciting the so-called waveguide mode. Upon excitation of the waveguide mode, an evanescent field with a penetration depth of 150 nm is created. The intensity of the evanescent filed decays exponentially with the distance from the sensor surface. The characteristic wavelength, at which the waveguide mode and evanescent field is created, is called the resonant wavelength. By changing the local refractive index inside the evanescent field, the resonant wavelength shifts to a new value. Proteins and living cells have a refractive index larger than that of the aqueous media; therefore, their adsorption or adhesion at the sensor surface shifts the local refractive index and detunes the resonant wavelength. The biosensor signal, the shift in the resonant wavelength, is monitored in real-time, thus not only the adhesion event and its magnitude, but also its kinetics can be followed. Each well of the microplate operates as separate sensors, therefore the microplate allows 96 or 384 parallel measurements by scanning the wavelength between 825 and 840 nm in every 3 s. The distribution of the resonant wavelength is imaged in each well by a high speed complementary metal oxide semiconductor camera^73,74^.

### Preparation of the biosensor surface with biomimetic coating for cell adhesion studies

The synthetic copolymers, poly(L-lysine)-*graft*-poly(ethylene glycol) (PLL-*g*-PEG, [PLL(20)-*g*(3.5)-PEG(2)]) (hereafter PP) and its RGD-functionalized counterpart, PLL-*g*-PEG/PEGGGGGYGRGDSP (PLL-*g*-PEG-RGD-12% [PLL(20)-*g*(3.5)-PEG(2.3)/PEG(3.4)-RGD]) (hereafter PPR) were obtained as powder from SuSoS AG (Dübendorf, Switzerland) and were stored at −20 °C until use. The stock solutions of 1.0 mg/ml PP and PPR were prepared in 10 mM HEPES (pH 7.4) and sterile-filtered. Surface coating with different concentration of RGD-motifs and PLL-*g*-PEG (hereafter PP:PPR) was created by mixing the two 1 mg/ml stock solutions in different ratios, than 50 µl (for 96-well plate) or 30 µl (for 384-well plate) of this mixture was added to the prewetted wells of Epic biosensor plate and incubated for 30 min at room temperature on the shaking machine. Reagent excess was removed by rinsing the surface three times with 20 mM HEPES HBSS, pH 7.4.

### Cell culture

The osteoblastic cell line MC3T3-E1 (93021013, Sigma-Aldrich Chemie GmbH, Schelldorf, Germany) was maintained in α-modified minimal essential medium (α-MEM, M4526, Sigma-Aldrich Chemie GmbH, Schelldorf, Germany), supplemented with 10% fetal bovine serum (Biowest SAS, France), 2 mM L-glutamine, 100 U/ml penicillin and 100 µg/ml streptomycin solution. Cells were cultured in a humidified atmosphere containing 5% CO_2_ at 37 °C. On reaching 80% confluence, cells were detached every 3–4 days using 0.05% (w/v) trypsin, 0.02% (w/v) EDTA solution and not used beyond passage 20.

For the experiments, MC3T3-E1 cells were removed from tissue culture dishes using a trypsin-EDTA. Trypsin digestion was terminated by the addition of completed medium and the harvested cells were centrifuged at 200 × g for 5 min. The cell pellet was resuspended in assay buffer (20 mM HEPES HBSS, pH 7.4) for serum-free conditions or in serum-containing cell culture medium.

### Epic cell adhesion assay on RGD-tuned surfaces

Coatings of a 96-well Corning Epic assay microplate with mixtures of PP and PPR synthetic copolymers at different PP:PPR ratio (v/v %, from 0 to 100% of PPR) were prepared as described earlier^26^. Upon surface modification, stable baselines were established in the wells with 50 µl of 20 mM HEPES HBSS, pH 7.4. MC3T3-E1 cells were harvested and 100 µl of cell suspension containing 8000 cells in assay buffer were added to the sensor wells. For buffer controls, all wells were coated with appropriate copolymers and then cell-free assay buffer was added. All measurements were replicated at least three times at room temperature. The averaged response of cell adhesion on the antifouling PP surface was used for background correction.

### Epic cell adhesion assay on glyphosate-containing surface

Prior to addition of the cell suspensions, a baseline was recorded in the wells of the Corning Epic cell assay microplate for 1 h with 25 μl of glyphosate solutions at different concentrations or in appropriate assay medium (as negative control), without previous surface modifications. Upon stable baselines had been established for all wells, the MC3T3-E1 cells were harvested and 25 µl of cell suspension containing 8000 cells in assay medium were added to the wells using an electronic 16-channel pipette Finnpipette™ Novus (Thermo Fisher Scientific, Waltham, MA, USA) in stepping mode. Biosensor responses were recorded for 1 h. All treatments were replicated at least three times within each experiment at room temperature, and experiments (with serial concentration series) were repeated 3–5 times. The averaged response of the cell-free solution was used for background correction.

### Epic cell adhesion inhibition assay

384-well Corning Epic assay microplate was coated with PP:PPR (1:1) as described above and then a baseline was recorded in the wells for 1 h in 20 μl 20 mM HEPES HBSS, pH 7.4. After the stable baselines had been established for all wells, the MC3T3-E1 cells were harvested and solutions containing glyphosate at varying concentrations were added to the cell suspension, resulting in a final cell density of 8000 cells/well. Then 30 μl of cell suspensions were seeded into the wells and biosensor responses were recorded for 1 h. Untreated MC3T3-E1 cells were used as negative control in the experiments. Reagent addition was performed using an electronic 16-channel pipette Finnpipette™ Novus in stepping mode. All treatments were replicated at least three times within each experiment at room temperature, and the whole experiments were repeated 3–5 times.

### Calculation of surface adsorbed mass of glyphosate from the Epic BT biosensor data

The resonant wavelength shift (Δλ (pm)) recorded by the biosensor can be transformed to surface adsorbed mass (ng/cm^2^) by using the calibration equation of Orgovan *et al*.^74^. It should be noted that, the equation in^32^ is valid for polyelectrolyte solutions with a refractive index increment of dn/dc = 0.1955 cm^3^/g. The refractive index values of glyphosate solutions were determined at room temperature using a Rudolph J157 table top refractometer (Rudolph Research Analytical, Hackettstwon, NJ, USA). The refractive index increment of glyphosate dn/dc obtained was 0.2051 cm^3^/g. Therefore, the adsorbed mass can be calculated from the Epic BT data by the following modified calibration equation:

6 $${\rm{\Delta }}M=0.296\,\frac{ng}{pm\cdot c{m}^{2}}\cdot {\rm{\Delta }}{\rm{\lambda }}$$

where ΔM is the surface adsorbed glyphosate mass (ng/cm^2^), and Δλ is the measured wavelength shift (in pm).

### Phase contrast microscopy

Following Epic measurements cells were visualized in microplates using a Zeiss Axio Observer.Z1 microscope (Carl Zeiss AG, Oberkochen, Germany) with a 20× objective. Phase contrast images were captured and analysed using the AxioVision software (Carl Zeiss AG).

### Competitive Epic integrin binding assay

The receptor binding assays were performed in 384-well Corning Epic assay microplates. The sensor surface was coated with 250 µg/ml PP:PPR (1:1) mixture in 10 mM HEPES pH 7.4 as above. Solutions of echistatin (to final concentrations of 20 and 40 nM), glyphosate (to final concentrations of 1.1 µM, 110 µM, 11 mM, and 22 mM), and tirofiban (to a final concentration of 500 nM) were added to the biosensor wells (20 µl/well). Upon stable baselines had been established for all wells in the Epic instrument, 20 µl of αvβ3 integrin (R&D Systems, Minneapolis, MN, #3050-AV) solution was added from a 384-well source plate to the biosensor microplate (at a final concentration of 4 µg/ml) using an electronic 16-channel pipette Finnpipette™ Novus and biosensor responses were recorded for 1 h at room temperature. Obtained biosensor data were corrected to the background (coated wells with buffer) and normalised to the averaged signal of the integrin binding without inhibitor (control).

### Competitive enzyme-linked immunosorbent assay

ELISAs were carried out in high-capacity 96-well microplates (Nunc, Roskilde, DK, #442404). Plates were coated overnight at 4 °C with 100 µl/well of 250 µg/ml PPR in 10 mM HEPES (pH 7.4). Thereafter, plates were washed three times with PBS-Tween buffer (137 mM NaCl, 2.7 mM KCl, 10 mM Na_2_HPO_4_, 2 mM KH_2_PO_4_, 0.01% Tween 20, pH 7.4) and blocked for 1 h at room temperature with 150 µl/well of Tris-BSA buffer (20 mM Tris-HCl, 150 mM NaCl, 1 mM CaCl_2_, 1 mM MgCl_2_, 1 mM MnCl_2_, pH 7.5, 1% BSA). Upon subsequent three washing steps with PBS-Tween buffer, soluble integrin αvβ3 (2.0 µg/ml) and integrin inhibitors in Tris-BSA buffer added in volumes of 50 µl/well each were incubated in the coated wells for 1 h at room temperature. Subsequently, the plates were washed three times with PBS-Tween buffer and then 100 µl/well of the primary antibody (mouse anti-human CD51/61, BD Biosciences) in Tris-BSA buffer at 2 µg/ml was added and incubated for 1 h at room temperature. After washing tree times with PBS-Tween, 100 µl/well of the secondary horseradish peroxidase (HRP)-conjugated antibody (anti-mouse IgG-HRP) in Tris-BSA buffer at 1 µg/ml was added and incubated for 1 h at room temperature. After three washing steps with PBS-Tween buffer, the detection of HRP was performed using 100 µl/well of 1.2 mM of hydrogen peroxide as a substrate with 1.2 mM of 3,3′,5,5′-tetramethylbenzidine as a chromophore in 0.5 M citrate buffer (pH 5.0). Upon sufficient colour development (after 10–60 min), the enzymatic reaction was stopped by the addition of 50 µl/well of 4 N sulphuric acid, and colour intensities in the wells were read at 450 nm in endpoint mode using a SpectraMax iD3 Multi-Mode Microplate Reader (Molecular Devices, San Jose, CA, USA). Obtained immunoassay data were corrected to the background (coated wells with buffer) and normalised to the averaged signal of the integrin binding without inhibitor (control).

### Statistical analysis

The shifts in the resonant wavelength, as the main signals detected in the Epic BT assay, were determined in at least three parallel treatments in each experiment, and all values are presented as mean ± SD. Statistical analyses were carried out for all treatments either on the Epic BT assay signals or on their values normalised to the negative control of the given experiment. Obtained data were analysed by using GraphPad Prism 6.0 (GraphPad Software, La Jolla, CA, USA) or Origin 8.5 (OriginLab Corp., Northampton, MA, USA). Concentration dependence was assessed by non-linear fit using sigmoid calibration curves on the basis of the four parameter logistic regression equation^75^. Statistical significance determination of the integrin binding results was performed with one-way ANOVA with statistical significance set to a p < 0.05.

## Data Availability

The data generated or analysed during this study are available from the corresponding author on reasonable request.
